# Radiofrequency Echographic Multi Spectrometry—A Novel Tool in the Diagnosis of Osteoporosis and Prediction of Fragility Fractures: A Systematic Review

**DOI:** 10.3390/diagnostics15050555

**Published:** 2025-02-25

**Authors:** Elena Icătoiu, Andreea-Iulia Vlădulescu-Trandafir, Laura-Maria Groșeanu, Florian Berghea, Claudia-Oana Cobilinschi, Claudia-Gabriela Potcovaru, Andra-Rodica Bălănescu, Violeta-Claudia Bojincă

**Affiliations:** 1 Department of Internal Medicine and Rheumatology, Sfanta Maria Clinical Hospital, 011172 Bucharest, Romania; elena.juganaru@drd.umfcd.ro (E.I.); maria.groseanu@umfcd.ro (L.-M.G.); florian.berghea@umfcd.ro (F.B.); claudia.cobilinschi@umfcd.ro (C.-O.C.); andra.balanescu@umfcd.ro (A.-R.B.); violeta.bojinca@umfcd.ro (V.-C.B.); 2 Faculty of Medicine, Carol Davila University of Medicine and Pharmacy, 020022 Bucharest, Romania; claudia-gabriela.potcovaru@drd.umfcd.ro; 3 Neuromuscular Rehabilitation Clinic Division, Teaching Hospital Bagdasar-Arseni, 041915 Bucharest, Romania

**Keywords:** REMS, DXA, comparison, accuracy, fracture risk, osteoporosis

## Abstract

**Background/Objectives:** Given the significant economic and social burden of osteoporosis, there is growing interest in developing an efficient alternative to the traditional dual-energy X-ray absorptiometry (DXA). Radiofrequency Echographic Multi Spectrometry (REMS) is an innovative, non-ionizing imaging technique that recently emerged as a viable tool to diagnose osteoporosis and estimate the fragility fracture risk. Nevertheless, its clinical use is still limited due to its novelty and continuing uncertainty of long-term performance. **Methods:** In order to evaluate the accuracy of the REMS, a systematic review of the English-language literature was conducted. Three databases were searched for relevant publications from 1 January 2015 until 1 December 2024 using the keyword combinations “(radiofrequency echographic multi spectrometry OR REMS) AND (dual-energy X-ray absorptiometry OR DXA)”. The initial search yielded 602 candidate articles. After screening the titles and abstracts following the eligibility criteria, 17 publications remained for full-text evaluation. **Results:** The reviewed studies demonstrated strong diagnostic agreement between REMS and DXA. Additionally, REMS showed enhanced diagnostic capabilities in cases where lumbar bone mineral density measurements by DXA were impaired by artifacts such as vertebral fractures, deformities, osteoarthritis, or vascular calcifications. REMS exhibited excellent intra-operator repeatability and precision, comparable to or exceeding the reported performance of DXA. The fragility score (FS), a parameter reflecting bone quality and structural integrity, effectively discriminated between fractured and non-fractured patients. Moreover, REMS proved to be a radiation-free option for bone health monitoring in radiation-sensitive populations or patients requiring frequent imaging to assess fracture risk. **Conclusions:** This current study underscores the robustness of REMS as a reliable method for diagnosing and monitoring osteoporosis and evaluating bone fragility via the FS. It also identifies critical knowledge gaps and emphasizes the need for further prospective studies to validate and expand the clinical applications of REMS across diverse patient populations.

## 1. Introduction

Osteoporosis has been defined as “a disease characterized by low bone mass and microarchitecture deterioration of the bone tissue, with a consequent increase in bone fragility and susceptibility to fracture” [1]. It is the most frequent bone disease worldwide, which manifests in one in three women and one in five men over the age of 50 [2]. Recent statistics indicate that up to 75 million people in Europe, the USA, and Japan are affected by osteoporosis, while many other cases are likely underdiagnosed [3]. Its prevalence is steadily growing due to population aging, making this condition increasingly recognized as a serious, worldwide public health concern.

Osteoporosis is usually a silent disease in the absence of fragility fractures, resulting in delayed diagnosis and treatment until complications have already occurred. Both a scarce awareness of the extension of the disease among the population and the limitations in terms of adequate techniques for the accurate assessment of fracture risk may contribute to the increasing personal and economic burden of this condition [3].

One-half of all Caucasian females and one-fifth of the males worldwide are estimated to experience an osteoporosis-related fracture at some point in their lives [4]. This generates significant disability and impaired quality of life; comparisons across diseases in terms of disability-adjusted life-years (DALYs) show that osteoporosis was outranked only by lung cancer, dementia, and ischemic heart disease [5,6]. In a prospective Polish study published in 2007, the annual mortality rate following a proximal femur fracture was 29.4% [7]. Moreover, the direct health costs associated with osteoporosis-related fractures proved to be extremely high in the year following the event, as outlined in a retrospective French cohort of individuals over 50 years of age [8].

Although bone architecture degeneration was considered an inevitable consequence of aging, nowadays, it is recognized as a serious and treatable disease with preventable complications, which requires medical monitoring, prompt diagnosis, and treatment [3].

Traditionally, DXA at the axial reference sites (lumbar spine and femoral neck) is deemed the gold standard for diagnosing osteoporosis. Given that it is relatively inexpensive with short scan times and low radiation exposure, it has maintained a privileged role in this field after its approval by the Food and Drug Administration (FDA) for clinical use in 1988 [9]. An incident X-ray passes through the patient’s body, and according to the properties of the crossed tissues, a degree of attenuation of the X-ray is measured by a detector [10]. Thus, a planar image is created, further serving to determine bone mineral density (BMD), which is expressed as grams per unit of area (g/cm^2^). The software also provides the T-score (the number of standard deviations (SDs) from the peak BMD of young women in the reference database) and the Z-score (the number of SDs from the mean BMD of age-matched women in the same standard reference database) [11]. The definition of osteoporosis, according to the World Health Organization (WHO), is based on the measured BMD of more than 2.5 standard deviations (T score < −2.5) below the mean value for young adults. However, the International Osteoporosis Foundation (IOF) reported that the limited accessibility of DXA examinations due to deficiency of densitometers or qualified personnel and the absence of reimbursement for associated costs are the main factors contributing to underdiagnosing osteoporosis in several European countries [12].

This has induced researchers to enhance their focus on osteoporosis management in order to develop more feasible procedures for an earlier and more accurate diagnosis in a simple and economically sustained way. Several complementary or alternative methods, such as Quantitative Computed Tomography (QCT), peripheral QCT (pQCT), high-resolution pQCT (HR-pQCT), Quantitative Ultrasonography (QUS), and Magnetic Resonance Imaging (MRI) have been considered [13]. Advanced software linked to HR-CT or HR-MRI can calculate bone strength, recognize fracture initiation sites and directions, and explore correlations between fracture risk and bone microarchitecture. Finite Element Analysis (FEA) is one such system that has been shown to predict fracture risk more accurately than BMD measured by DXA [14]. All these modalities have certain specific advantages; however, their application in clinical practice is constrained by various factors, which individually include radiation (CT technique), limited portability and availability for clinical use (pQCT, MRI), high costs, specific site planning for construction, and long time requirements for acquisition, which can result in motion artifacts (MRI) or operator/technical (ambient temperature)-dependent variations (QUS).

However, the emergence of REMS technology offers a novel approach, with potential advantages in the context of the above-mentioned limitations that are currently being faced. It is a portable radiation-free tool that uses raw, unfiltered native ultrasound (US) signals (radio-frequency US signals) for the evaluation of bone health status assessment and fracture risk prediction, while also being cost-effective [15]. This scan uses a 3.5 MHz convex US probe to emit US at the target sites (lumbar spine and proximal femur). The back-scattered waveforms generated are then captured by the receiver and reconstructed into a B-mode image [16]. The software identifies the bone interfaces within the regions of interest (ROIs) and analyzes the back-diffused US signals. The recorded signal spectra are compared against reference spectral models matched by gender, age, site, and body mass index (BMI) from a dedicated database [17]. From this analysis, quantitative parameters such as BMD values, T-scores, and Z-scores are calculated, enabling the classification of bone as normal, osteopenic, or osteoporotic. In addition to these measures, REMS provides a bone quality parameter, the FS, which assesses skeletal microarchitecture independently of BMD. This score ranges from 0 (normal) to 100 (maximum fragility of a bone structure) and is used to generate measures of fragility fracture risk over a five-year time interval [18].

REMS was already validated in a few clinical trials and approved by the FDA in October 2018, while the European Society for Clinical and Economic Aspects of Osteoporosis and Musculoskeletal Diseases (ESCEO) asserted it as a valuable tool for the diagnosis of osteoporosis and fracture risk prediction. Fuggle et al. [16] summarized ESCEO expert opinions on the clinical implementation of REMS based on the latest evidence. These opinions were structured using a Grading of Recommendations, Assessment, Development, and Evaluations (GRADE) framework. Five recommendations, particularly those addressing the use of REMS in specific populations including frail patients, received a grading of “strongly in favor of”. Meanwhile, three recommendations—regarding its ability to predict the risk of incident fracture, provide short-term monitoring, and overcome bone artifacts—were assessed as “weakly in favor of”. Against this backdrop, the novelty and broad potential that REMS offers over established methods of diagnosis keep it a subject of active debate. It elicits a mix of skepticism and cautious optimism from the scientific community, which may have restrained its widespread clinical adoption so far.

In this study, we aim to comprehensively review the existing literature on the performance of REMS in the diagnosis of osteoporosis and discrimination of patients at risk of incident fragility fractures compared to DXA. By synthesizing findings from various research studies, we seek to elucidate the strengths and limitations of REMS as an innovative diagnostic method in the field. Particular emphasis will be placed on the potential of REMS to identify parameters beyond BMD that reflect the qualitative and structural characteristics of the bone [19]. Our objective is to offer insights that support clinical practice and guide future research endeavors in osteoporosis assessment and management.

## 2. Materials and Methods

A systematic review of the English-language literature from the last 10 years on the accuracy of the REMS technique in the diagnosis of osteoporosis and fracture risk prediction was performed according to the guidelines and recommendations from the Preferred Reporting Items for Systematic Reviews and Meta-Analysis Checklist (PRISMA). The protocol for this review has been registered in PROSPERO with the identifier CRD42025634785.

### 2.1. Research Question and Search Strategy

The research question was formulated using the Population, Intervention, Comparison, and Outcome (PICO) method. The population was represented by adults with a medical indication for screening for osteoporosis (including age, postmenopausal status, prolonged exposure to medication predisposing to osteoporosis, history of low trauma fracture, risk factors for secondary osteoporosis, or other risk factors appreciated by the attending physician); intervention was represented by the usage of the REMS novel technology (while the comparator group was examined with the actual gold standard method—DXA). The outcome was defined by the validity of REMS to estimate bone parameters in patients screened for osteoporosis, with the effect measured by the percentage, sensitivity, specificity, area under the curve (AUC), or correlation coefficient.

A comprehensive search was conducted using the Web of Science, Pub Med, and Science Direct library databases to identify relevant publications from 1 January 2015 until 1 December 2024. The search strategy employed the following criteria: “(radiofrequency echographic multi spectrometry OR REMS) AND (dual-energy X-ray absorptiometry OR DXA)”. Although the search included all publications within this timeframe, it was noted that no studies using REMS technology were identified before 2019. Consequently, while earlier studies were considered, only those explicitly using the REMS designation were included to ensure consistency in terminology.

**Figure 1:**
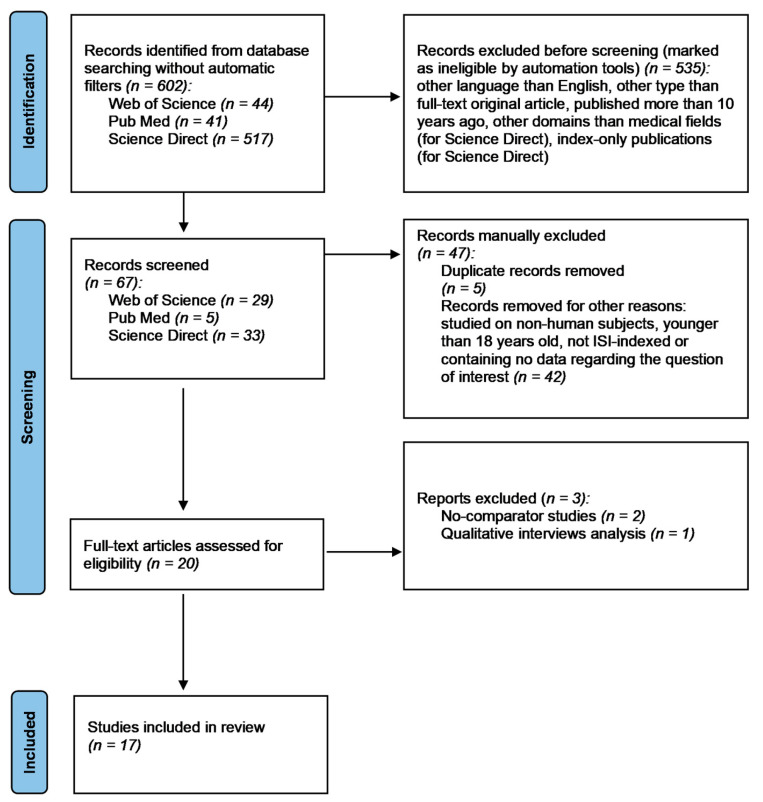
PRISMA flow diagram of the study selection process.

The search yielded a total of 602 candidate articles before applying any automatic filters (44 from Web of Science, 41 from Pub Med, and 517 from Science Direct). The selection process is summarized in Figure 1.

After applying filters for language (English), publication type (original articles), date range (2015 to the date of the search), and medical field (for Science Direct) and excluding the index-only publications in Science Direct, a total of 67 articles were retained. Five duplicates were manually removed and the rest of the 62 publications underwent initial title screening, followed by abstract review by two independent reviewers. After excluding papers that were not indexed in the Institute for Scientific Information (ISI), papers with different outcomes than the one of interest, papers without comparators, and papers studying non-human subjects or populations younger than 18 years old, 17 articles met the inclusion criteria and remained for full-text evaluation.

### 2.2. Inclusion, Exclusion Criteria and Selection of Studies

The following inclusion criteria were applied: (i) original articles designed as randomized clinical trials, observational prospective or retrospective clinical studies, or cross-sectional or case-control studies; (ii) publication date after January 2015; (iii) English language; and (iv) clearly comparing the performance of REMS and DXA. Non-human or pediatric studies, other types of publications than the ones mentioned above (such as conference abstracts, case reports, reviews, meta-analyses, or letters to editors), and papers written in a language different from English were excluded. We also removed studies lacking data of interest (performance metrics including accuracy, sensitivity, and specificity of the assessed technology) or focusing on different outcomes (e.g., cost-minimization analysis using structured qualitative interviews).

### 2.3. Data Extraction

After evaluating the identified articles in the three main databases and excluding duplicates, two reviewers independently screened the titles and abstracts following the same eligibility criteria based on the developed PICO. The Rayyan platform [20] was used for the title and abstract screening process.

Potentially relevant articles of interest were further considered for full-text analysis. Any differences in opinion were settled through consultation with a third author.

A self-made data extraction table (in Microsoft Excel Worksheet; Microsoft Office Professional Plus 2019) was used in order to synthesize the relevant data extracted from the studies, including the first author and year of publication, geographic region, study period, study design, sample size, average age of participants, patient characteristics, type of DXA machine, reported outcome measures, results, and comments.

### 2.4. Risk of Bias Assessment

Two authors independently assessed the risk of bias in the included studies using the Critical Appraisal Checklist (CASP) specially designed for each type of publication. This consisted of a set of questions focusing on different methodological aspects of the studies, which were evaluated through a ‘yes/no/can’t tell’ scale. Disagreements between authors were discussed and resolved through consensus. Table A1 and Table A2 in Appendix A illustrate the characteristics of the included articles.

### 2.5. Strategy for Data Synthesis

The included studies were summarized in a table format to include details on the study design, patient characteristics, reported outcomes, and quality rating (Table A3, Appendix B). Since studies were expected to be heterogeneous in terms of publication type, quality, interventions, and outcomes, a narrative synthesis was performed using text and tables in order to encapsulate the study characteristics and findings.

## 3. Results

### 3.1. Study Characteristics

This systematic review incorporates 17 studies focusing on the understanding of the capabilities of the REMS technique for the diagnosis of osteoporosis. These analyzed studies span various global regions, including Europe (Italy, Poland, Spain, Belgium, and the United Kingdom), Asia (Japan), and America (Brazil), but the majority are conducted on European populations. There was a total of 11,664 patients aged between 30 and 90 years, with females (9314) representing the majority (79.85%). Menopause status, BMI, age, and comorbidities were important confounding factors considered in almost all the studies.

All the selected studies were published between 2019 and 2024, with the majority (11 of them) during the last three years. This reflects the growing interest and attention this topic has recently gained within the scientific community.

Regarding the study design, two studies were prospective cohorts, while all the rest were cross-sectional studies. The prospective studies had a mean duration of five years.

Osteoporosis was defined in all the included studies according to the definition of the WHO, thus implying a DXA-measured BMD of more than 2.5 standard deviations (T score < −2.5) below the mean value for young adults in the reference database. Moreover, a T-score between −1 and −2.5 enabled the classification of the patients as “osteopenic”. All DXA scans were reported as performed according to the standard clinical routine procedures, using Hologic or GE Lunar densitometers. DXA output data were assumed to be the gold standard reference and were further compared with the results obtained using REMS to assess the latter technology’s performance.

In all the selected studies, REMS scans were performed with a dedicated US device (EchoStation, Echolight Spa, Lecce, Italy), equipped with a 3.5 MHz transducer and used as recommended by the manufacturer. The axial reference sites (proximal femur and/or lumbar spine) were assessed, enabling the calculation of BMD, T-, and Z-scores for each site.

Table A3 in Appendix B summarizes some paramount information extracted from the selected studies, as described above.

### 3.2. Comparative Diagnostic Performance of REMS and DXA in Bone Health Assessment

The first study focusing on REMS validity was an Italian multicenter cross-sectional study following 1914 postmenopausal women who underwent spinal and/or femoral DXA and REMS scans. Analysis of the values using the Bland–Altman plots showed good agreement between the two tests with an average difference in BMD of −0.004 ± 0.088 g/cm^2^ for the spine (r = 0.94, *p* < 0.001) and −0.006 ± 0.076 g/cm^2^ for the femur (r = 0.93, *p* < 0.001) [15]. The diagnostic concordance was 88.8% (Cohen’s kappa coefficient (k) = 0.824, *p* < 0.001) for the lumbar spine (LS) and 88.2% (k = 0.794, *p* < 0.001) for the femoral neck (FN), respectively. The diagnostic concordance reached 97.4% for LS and 98.0% for FN if a 0.3 tolerance on the borderline T-score was accepted.

These preliminary findings were further verified in a larger series of female Caucasian patients in a study by Cortet et al. This study identified a diagnostic concordance of about 86% between REMS and DXA both in the spine and the proximal femur [21]. Also, a prospective study conducted by Adami et al. [22] revealed similar results. The diagnostic agreement with DXA was 84.8% for the vertebral site and 84.2% for the femoral site, with a Pearson’s correlation coefficient r = 0.92 (*p* < 0.001). The diagnostic agreement for the three groups (osteoporosis, osteopenia, and healthy) was very good, as expressed by Cohen’s coefficient k = 0.8. The results are also in line with those presented by Nowakowska-Plaza et al. [7], who obtained diagnostic agreement rates of 82.2% (k = 0.611) in the LS and 84.8% (k = 0.667) in the FN in a Polish cohort. REMS was used in Latin American patients with the same accuracy of diagnosis [23].

Slightly weaker correlations between BMD-DXA and BMD-REMS at all skeletal sites were found in special cohorts of patients. These discrepancies may be at least partially attributed to inherent DXA inaccuracies, which will be discussed in more detail later in the text. For example, patients diagnosed with osteogenesis imperfecta showed the following results: r = 0.54, *p* < 0.01 for the FN, r = 0.65, *p* < 0.01 for the total hip (TH), and r = 0.35, *p* < 0.05 for the LS [24]. In patients with chronic kidney disease undergoing renal replacement therapy, the rate of diagnostic agreement ranged from fair for the LS (k = 0.321, *p* = 0.026) to moderate for the FN (k = 0.445, *p* < 0.01) and substantial for the TH (k = 0.784, *p* < 0.01) [25]. Specific details about the accuracy of REMS in these particular backgrounds will be discussed in the sections below.

Unanticipated results are provided by Lalli et al. [26], who reported weaker diagnostic concordance between the two techniques. The rate of agreement was minimal in patients with primary osteoporosis (k = 0.31, *p* = 0.001) and lacked statistical significance in disuse-related osteoporosis. The discrepancy between these findings and the previous data might be explained by differences in age, gender, or disease severity between the samples included. Moreover, for patients with disuse-related osteoporosis after spinal cord injury, the well-known alterations of muscle and fat tissues could also play a role in the reflection and attenuation of the US beam. Also, the sample was relatively small, which could have biased the results.

Table A3 in Appendix B provides a summary of the diagnostic agreement rates and correlations between REMS and DXA.

#### 3.2.1. Sensitivity and Specificity in Detecting Osteoporosis

In the study of Di Paola evaluating the performance of REMS [15], this technique proved a sensitivity of 91.5% at the FN and 91.7% at LS, while the specificity was 91.8% at the FN and 92% at the LS. The researchers emphasized that the REMS approach can result in great diagnostic outcomes granted that guidelines and recommendations are scrupulously followed. However, the significant number of REMS reports excluded because of operator-dependent errors must be considered as a possible bias when interpreting the results. Even so, its sensitivity and specificity were persistently high in the “real life scenario” when REMS reports containing errors were not excluded (sensitivity of 81% for the LS and 81.7% for the FN; specificity of 84.3% for the LS and 89.7% for the FN).

Later, the sensitivity and specificity of REMS detected in the study of Adami et al. evaluating men were 90.1% and 93.6% using the LS scans and 90.9% and 94.6% using the FN [27]. The results were comparable for the Latin cohort, where the sensitivity ranged between 84 and 92.6%, while the specificity had values between 93.5–94.6% [23].

The data on sensitivity and specificity reported in all the papers included are summarized in Table A3 in Appendix B.

#### 3.2.2. Reliability and Reproducibility of Results (REMS Intra- and Inter-Operator Repeatability)

The preliminary work on REMS performance in 2019 provided the first results regarding the precision and repeatability of outcomes of the technique [15]. Precision, expressed as the root-mean-square coefficient of variation (RMS-CV), was 0.38% for the LS, with a least significant change (LSC) value of 1.05%, and 0.32% for the FN, with an LSC of 0.88%. Analogously, repeatability RMS-CV was found to be 0.54% for the LS and 0.48% for the FN with corresponding LSC values of 1.5% and 1.33%. These values are superior to the average values reported in the literature for DXA, which indicate an RMS-CV of 2.02% for the LS and 1.29% for the FN, along with an LSC of 5.6% for the LS and 3.56% for the FN [28].

Adami et al. [27] outlined intra-operator repeatability (short-term precision) of 0.4% expressed as the RMS-CV for the LS and 0.34% for the FN, with an LSC of 1.1%, CI of 95%, and inter-operator variability of 0.57%, LSC = 1.57% for the LS and 0.52%, LSC = 1.43% for the FN. Intra-operator repeatability was determined based on an experienced operator’s performance who performed two consecutive measurements on the first 32 patients. Inter-operator repeatability implied two consecutive measurements performed by two operators on the same 32 patients. Similar results were found in further studies [29,30]. Amorim et al. [23] reported that the intra-operator precision error (RMS-CV) was 0.47% in the spine (LSC = 1.29%) and 0.32% at the femoral site (LSC = 0.89%). The inter-operator variability yielded RMS-CV error and LSC values of 0.55% and 1.52% for the LS and 0.51% and 1.40% for the proximal femur, respectively.

Intra- and inter-operator repeatability were also evaluated for the fragility score performance [31] and were estimated at 0.49% and 0.43% for the LS and 0.73% and 0.64% for the FN, respectively, at a 95% confidence interval.

#### 3.2.3. The Impact of Demographic Variations on REMS Diagnostic Accuracy

The diagnostic accuracy of REMS has been insufficiently investigated in specific populations stratified by demographic factors such as gender, age, BMI, or ethnicity. Nevertheless, these demographic characteristics could significantly influence the precise assessment of bone health.

- Osteoporosis in Males

While osteoporosis is more prevalent in females, with a global sex ratio of approximately 4:1 [27], males account for 30–40% of new osteoporotic fractures [32]. Furthermore, mortality rates following major osteoporotic fractures are higher in males compared to females [33], highlighting the critical need for focused medical intervention in this population. A multicenter cross-sectional study examined the performance of REMS relative to DXA in Caucasian men who had been prescribed DXA scans [27]. The study adhered to standardized protocols and reported high correlations between T-scores from REMS and DXA at both the LS (r = 0.91, *p* < 0.001) and FN (r = 0.90, *p* < 0.001). Diagnostic concordance across the three diagnostic categories (osteoporosis, osteopenia, and healthy) was substantial, with agreement rates of 82.7% (k = 0.71, *p* < 0.001) for the LS and 81.8% (k = 0.71, *p* < 0.001) for the FN [27]. Bland–Altman plots (−0.06 ± 0.60 g/cm^2^ for the LS and −0.07 ± 0.44 g/cm^2^ for the FN) confirmed the absence of systematic biases between REMS and DXA T-scores, reinforcing the potential applicability of REMS in male patients.

- Age

The effect of a patient’s age on the diagnostic accuracy of REMS was studied in detail in a large European cohort of 4307 female patients. The age-stratified analysis showed that diagnostic concordance between DXA and REMS fluctuated between 81.4 and 92% [21] and was inversely associated with age.

Adami et al. [27] demonstrated through a linear regression analysis that the strong correlation between DXA and REMS T-scores persisted when patients were grouped by age (<65 years vs. ≥65 years old). Notably, older patients exhibited higher correlation coefficients for lumbar scans (r = 0.94, *p* < 0.001 for ≥65 years old; r = 0.89, *p* < 0.001, for <65 years), while FN scans showed similar correlation levels across both groups (r = 0.9, *p* < 0.001). Sensitivity for REMS was higher in younger patients (91.4% and 85% for lumbar scans; 100% and 81.3% for femoral scans) compared to older patients, with specificity following a similar pattern (94.5% and 92% for lumbar scans; 95.7% and 92.5% for femoral scans). This finding needs to be cautiously interpreted though since younger populations experience a lower prevalence of osteoporosis, so a smaller number of total cases will be diagnosed. Despite these differences, diagnostic agreement across the three classes remained consistent regardless of age. In accordance with these results, Nowakowska-Plaza et al. disclosed a strong correlation between REMS and DXA scores both in young (r = 0.781, *p* < 0.001) and elderly patients (r = 0.834, *p* < 0.001) [7].

- Body Mass Index

Cortet et al. [34] highlighted excellent diagnostic agreement between REMS and DXA across different BMI categories (r = 0.946 for BMI < 18.5 kg/m^2^; r = 0.946 for BMI ≥ 18.5–25 kg/m^2^; r = 0.930 for BMI ≥ 25 kg/m^2^). Sensitivity slightly declined with increasing BMI (97.5%, 92.6%, and 86.5% for the respective categories), while specificity remained high. The results were validated against data from a Polish cohort, indicating a robust correlation between REMS and DXA scores, which remained consistently strong regardless of BMI variations (r = 0.858, *p* < 0.001 for a BMI < 25 kg/m^2^; r = 0.776, *p* < 0.001 for a BMI ≥ 25 kg/m^2^) [7]. However, in a cohort study conducted in Japan [35], where patients were divided into subgroups based on the discrepancies in DXA and REMS results, a notable disparity was observed in BMI. Patients with greater score discrepancies between the two technologies had a higher BMI compared to those with smaller differences (23.55 vs. 21.96 kg/m^2^; *p* = 0.045).

Due to manufacturer constraints, REMS has not been validated for individuals with severe obesity (BMI > 40 kg/m^2^), and no published data currently address this population.

#### 3.2.4. Exploring Errors in REMS and DXA Reports

Di Paola et al. [15] underscored in their paper that strong agreement exists between REMS and DXA-based diagnoses when both examinations are performed strictly according to the guidelines. Even so, the results were derived after excluding DXA and REMS reports that did not meet the required standards. Expressly, 5% of the patients in the lumbar groups and 3.6% in the femoral groups were excluded due to DXA errors, while 18% in the lumbar group and 12.5% in the femoral group were excluded for irrecoverable REMS errors. The difference in handling errors between the two techniques stems from the fact that DXA permits re-analysis of erroneous reports, whereas REMS does not [15]. Adami et al. [27] explored the adhesion of the operators to the strict guidelines for DXA and REMS scans and excluded the errors identified in both categories after all reports were independently checked by two experienced operators. The most common errors encountered in DXA were represented by inaccurate patient positioning, analysis pitfalls, artifacts, or mistakes in the demographic characteristics of the patients, issues frequently reported in many other papers assessing the quality of measurement [19,27,36]. These errors led to the exclusion of 8.8% of the lumbar scans and 7.5% of the femoral scans. In contrast, the most frequent REMS errors were represented by incorrect acquisition parameter settings, such as suboptimal adjustments of focus/scan depth and unsynchronized movement of the probe with the audible instructions provided by the software [15,27,37]. It is noteworthy to mention that REMS employs an automatic algorithm that independently excludes non-diagnostic scans.

### 3.3. Evaluating Bone Quality and Structural Integrity: The Role of Fragility Score in Predicting Fragility Fractures

REMS-derived parameters have proven to be effective predictors of fragility fracture risk compared to DXA [22]. A key clinical finding was that REMS demonstrated superior performance in identifying fragility fractures using the lumbar T-scores (OR = 2.6, AUC = 0.66) compared to the DXA-assessed T-scores (OR = 1.7, AUC = 0.61). The sensitivity and specificity of REMS T-scores for detecting fragility fractures were 65.1% and 57.7% at the vertebral site vs. 40.2% and 79.9% at the FN. In contrast, DXA exhibited slightly lower sensitivity and specificity for both sites (57.1% and 56.3% at the LS; 42.3% and 79.3% at the FN). Notably, REMS showed higher positive and comparable negative predictive values at the LS. No significant differences between the two methods were observed at the FN. Although the results are encouraging, the small sample sizes for each fracture type might represent a bias that requires consideration. However, the findings were verified in a large European multicenter cohort [21] and REMS T-scores performed better than DXA in discriminating between fractured and non-fractured patients aged 30–90 years (AUC = 0.632 for DXA; AUC = 0.683 for REMS, *p* < 0.001 at the femur; AUC = 0.603 for DXA; AUC = 0.640 for REMS, *p* = 0.0002).

In a 5-year prospective study [31], Pisani et al. evaluated the FS parameter derived from REMS. The paper emphasized the superior predictive capacity of the FS over BMD T-scores in identifying patients at risk of fragility fractures. In women, the FS cutoff values indicated a 9-fold higher risk for fragility fractures in the LS and a 6-fold higher risk in the hip compared to any BMD measurements. The LS-FS achieved an AUC of 0.811, outperforming both REMS (AUC = 0.709, *p* < 0.0001) and DXA BMD T-score (AUC = 0.678, *p* < 0.0001), for distinguishing women with and without incident fracture (*p* = 0.003 and *p* < 0.0001, respectively). Similarly, in men, the FS indicated a 9.5-fold increased risk of fragility fractures in the LS and an 8.3-fold increased risk of hip fractures. The LS-FS achieved an AUC of 0.780, which was significantly higher than those of the REMS (AUC = 0.652, *p* < 0.0001) and DXA BMD T-score (AUC = 0.635, *p* = 0.0001), for distinguishing men with and without incident fractures (*p* = 0.007 and *p* = 0.002, respectively). Similar results were observed for the FN in both genders, with the age-adjusted AUC of the FS showing a consistent trend. Sensitivity and specificity for FS varied between 70 and 72.4% in women and 73.2 and 77.9% in men across both measurement sites.

The present findings seem to be consistent with the research Lalli et al. conducted [26], which demonstrated significant differences in FS between fractured and non-fractured patients in cases of both primary and disuse-related osteoporosis (*p* = 0.0001; *p* = 0.01).

#### Fracture Risk Assessment in Osteoporosis: Insights from REMS, DXA, TBS, and FRAX Scores

REMS validity in fracture prediction necessitates a comparison with established tools such as the trabecular bone score (TBS) or Fracture Risk Assessment (FRAX) tool, which are reported to be capable of independently predicting risk beyond BMD and clinical risk factors [38,39]. Nevertheless, the field has yet to be explored sufficiently.

Bone health in patients diagnosed with clinical or genetic osteogenesis imperfecta (OI) was assessed using REMS, DXA, and TBS [24]. TBS was calculated using the standard DXA scan of the antero-posterior LS. While DXA revealed no significant differences in BMD among the patients with different types of OI, both TBS and REMS detected lower LS values in patients diagnosed with types III and IV compared to type I (*p* < 0.05). Additionally, the study underlined the superior correlation of BMD-REMS with TBS at the LS (r = 0.648, *p* < 0.001) compared to BMD-DXA (r = 0.284, *p* = 0.05).

Contradicting these findings, Fassio et al. [25] stated that TBS correlated with the DXA-T-scores across all evaluated sites, including the antero-posterior LS (R^2^ = 0.31, *p* < 0.01), latero-lateral LS (R^2^ = 0.21, *p* < 0.01), FN (R^2^ = 0.27, *p* < 0.01), and TH (R^2^ = 0.31, *p* < 0.01). However, no significant correlation was observed between TBS values and REMS at any site.

The utility of DXA and REMS for calculating FRAX and the FRAX- Derived Fracture Risk Assessment (DeFra) was also evaluated. Both technologies produced comparable results, with no statistically significant differences between the fracture risk estimates, suggesting equivalence in their application for risk assessment [25].

Furthermore, Lalli et al. [26] reported significant positive correlations in patients with primary osteoporosis without major fragility fractures between the REMS-FS and the FRAX-derived 10-year probability of both major fractures (R = 0.65, *p* = 0.0001) and hip fracture (R = 0.62; *p* = 0.0001).

### 3.4. Clinical Applicability of REMS Across Diverse Patient Populations

#### 3.4.1. Radiation-Free Bone Assessment: Advantages of REMS for Pregnant Women, Breastfeeding Women, Children, and Longitudinal Monitoring of Patients at High Risk of Fracture

The REMS technology represents a particularly significant advancement for specific populations where traditional methods, such as DXA, pose limitations due to their reliance on ionizing radiation. As a radiation-free method, REMS offers a safe and reliable option for monitoring bone health across diverse patient groups. These include children, adolescents, individuals of reproductive age, pregnant or breastfeeding women, and patients with conditions requiring frequent radiological evaluations due to high fracture risk.

Although pediatric patients fall outside the scope of this study, the potential applications of REMS in this population are notable, particularly in disorders affecting bone metabolism with early onset during childhood. For example, OI serves as a representative condition and is discussed in detail below.

During pregnancy, where data on osteoporosis remain limited, auxiliary diagnostic methods are utilized to estimate lower BMD values [40]. However, conducting comparative studies between DXA and REMS presents significant ethical challenges.

Moreover, chronic illnesses that render young female patients even more vulnerable to osteoporosis warrant particular attention. One notable example, particularly assessed in the included articles [41], is restrictive anorexia nervosa (AN), a psychiatric disorder characterized by undernutrition and endocrine dysfunctions, primarily affecting adolescent and childbearing-age women. AN is associated with low BMD and frequently complicated with fragility fractures due to both cortical and trabecular bone changes [42]. Preliminary work on young women diagnosed with AN aimed to determine the usefulness of REMS for the evaluation of bone status in this population [41]. Both REMS and DXA scans revealed significantly lower BMD values across all measurement sites in patients with AN compared to controls (*p* < 0.01). Strong agreement between BMD Z-scores by REMS and DXA at the LS and TH was noted, confirmed by Bland–Altman analysis (average differences of 0.012 ± 0.350 g/cm^2^ for the LS and −0.080 ± 0.288 g/cm^2^ for the TH) [43]. However, REMS-measured Z-scores at the FN were significantly lower (*p* < 0.05) than those obtained by DXA [41]. Additionally, AN patients with prior vertebral fragility fractures presented significantly lower REMS-measured BMD at the TH compared to those without fractures (*p* < 0.05).

#### 3.4.2. REMS: A Breakthrough Technique for Overcoming DXA-Artifacts in the Lumbar Spine?

Osteoarthritis (OA) poses a significant diagnostic challenge in identifying osteoporosis, as the presence of OA-related structural changes can obscure osteoporosis detection, particularly with current gold-standard diagnostic methods.

A study conducted by Caffarelli et al. in 2022 [37], involving a cohort of 159 Caucasian women (mean age 66.2 ± 11.6 years), with radiologically diagnosed OA/vertebral fractures in the LS, demonstrated the potential of REMS in overcoming these limitations. In this study group, REMS identified osteoporosis in 35.1% of cases, compared to just 9.3% identified using DXA. In addition, the proportion of patients with vertebral fractures classified as osteoporotic was significantly higher with REMS compared to DXA (58.7% vs. 23.3%, respectively).

Furthermore, a recent study from Japan highlighted the advantages of REMS in addressing artifacts caused by calcifications, prostheses, osteophytes, and vertebral fractures [35]. This study employed semi-quantitative analysis using validated scores to assess the severity of vertebral fractures, hip OA deformities, and aortic calcifications. Consistent with expectations, the greatest discrepancies in BMD and T-scores between DXA and REMS were observed in the LS, where REMS recorded a BMD of 0.623 c/cm^2^ and a T-score of −3.2, compared to DXA-BMD of 0.966 g/cm^2^ and DXA-T-score of −1.3 (*p* < 0.001) [35]. Similar trends were remarked at other measurement sites, with patients exhibiting significant discrepancies between DXA and REMS scores also demonstrating a higher incidence of multiple vertebral fractures and vascular calcifications.

The DEMETER study corroborated these findings [29], showing that the T-scores at the LS obtained by DXA were consistently higher with respect to those generated by REMS, regardless of OA severity as assessed by the Kellgren–Lawrence (K-L) grading system. Consequently, REMS identified a greater percentage of patients with osteopenia or osteoporosis compared to DXA (30.5% and 52.0% vs. 6.0% and 34.5%, respectively, *p* < 0.001). Notably, the differences between the DXA and REMS scores were more pronounced in patients with severe LS-OA (K-L score = 0/1, r = 0.98, *p* < 0.0001; K/L score = 2, r = 0.85, *p* < 0.001; K/L score = 3, r = 0.45, *p* < 0.01, respectively). Another major finding is that hip BMD by DXA also increased with the severity of LS-OA, while the REMS-derived BMD values at both the FN and TH decreased as the grade of LS-OA increased.

#### 3.4.3. Expanding the Applicability of REMS to Patients with Chronic Kidney Disease, Type 2 Diabetes Mellitus, and Other Conditions Involving Altered Bone Metabolism

Artifacts significantly impact BMD estimates, a phenomenon also observed in patients with chronic kidney disease (CKD) undergoing hemodialysis or peritoneal dialysis, where mineral bone metabolism is frequently disturbed [44]. In these patients, DXA-based BMD evaluations face substantial limitations due to factors such as aortic and ligamentous calcifications, degenerative changes, fractures, or scoliosis, all of which can result in overestimated BMD values.

A study by Fassio et al. [25] evaluated bone status in patients undergoing peritoneal dialysis. The extent of the aortic calcifications was graded using the Kaupilla score (0–24) [45]. This study identified significant positive correlations (*p* < 0.01) between the total calcification scores and the differences in T-scores derived from DXA in an antero-posterior and latero-lateral LS exposure. Conversely, latero-lateral DXA and REMS T-scores at all sites exhibited negative correlations with the aortic calcifications scores, while these results were not confirmed in the antero-posterior LS. No statistical differences were observed between DXA and REMS T-scores at the FN and TH. Cohen’s coefficients for osteoporosis revealed fair diagnostic agreement for the LS (k = 0.321, *p* = 0.026), moderate agreement at the FN (k = 0.445, *p* < 0.01), and substantial agreement at the TH (k = 0.784, *p* < 0.01) [25]. These findings are in line with another study involving kidney transplant recipients [46], where modest correlations between DXA and REMS were noted at the LS (r = 0.4, *p* < 0.01) and stronger at the FN (r = 0.7, *p* < 0.0001). REMS identified a higher prevalence of osteopenia or osteoporosis at the LS (23% and 65%, respectively) than DXA (20%, 45%, *p* < 0.05).

Another critical gap in bone status assessment is represented by the impaired osseous quality in type-2 diabetes mellitus (T2DM) associated with a consequent reduction in bone strength [19]. This area of focus was recently addressed in a cross-sectional study by Caffarelli et al. involving 88 Caucasian postmenopausal women with longstanding T2DM [19]. REMS demonstrated its utility by detecting microarchitectural modifications of the bone tissue in T2DM women with prior fragility fractures, where LS-BMD by REMS was significantly lower with respect to patients without fractures. Moreover, BMD T-scores derived from REMS were significantly lower than those from DXA both at the LS (*p* < 0.01) and FN (*p* < 0.05), resulting in a greater proportion of T2DM patients classified as osteoporotic by REMS (47.0% vs. 28.0%, respectively). Comparisons with age- and sex-matched controls revealed significantly higher DXA-measured BMD values at both the LS and TH in T2DM patients, potentially reflecting the association between T2DM and OA beyond weight-related factors [47].

Other endocrinopathies, such as acromegaly, also complicate bone status assessment due to their complex effects on bone metabolism, resulting in increased cortical bone thickness, cortical porosity, and trabecular impairment [48]. This leads to a great risk of fractures and implies that BMD has major limitations in the evaluation of these patients. Rolla et al. found no significant differences in bone parameters (BMD, T-scores, and Z-scores) measured by REMS and DXA at the LS and hip in acromegaly patients compared to controls [36]. No differences were noted between well-controlled and surgically cured patients, emphasizing BMD’s limited reliability in this population [49]. Correlations between REMS and DXA in acromegaly patients were weaker than reported for other groups (r = 0.482, *p* = 0.011 for LS and r = 0.431, *p* = 0.018 for FN), likely reflecting the unique challenges posed by cortical bone abnormalities and possibly a high prevalence of osteoarthritis in these patients.

As was already stated earlier in this paper, the application of REMS has also been explored in the context of OI, a prototypical hereditary connective tissue characterized by qualitative and quantitative abnormalities in bone collagen, osteoblast differentiation, and bone mineralization, thus contributing to low trabecular and cortical thickness, higher intracortical porosity, and altered bone biomechanics [50]. Studies have demonstrated that BMD values, as assessed by both DXA and REMS, are significantly lower in OI patients than controls [24]. However, notable differences were observed when comparing parameters obtained through the two technologies. Specifically, the TH-BMD values assessed by REMS were significantly higher than those by DXA (*p* < 0.05), while LS Z-scores determined by REMS were lower than those by DXA.

## 4. Discussion

This systematic literature review provides valuable insight into the performance of the REMS technology, which has recently gained recognition as a promising imaging modality for osteoporosis diagnosis and fracture risk stratification. The principal conclusions of this study can be summarized as follows: (i) REMS demonstrates diagnostic accuracy comparable to DXA, with strong diagnostic concordance (63.6–90%) [15,35] as well as high sensitivity (70–91.7%) and specificity(73.2–95.5%) [15,23,31]; (ii) in patients with chronic disorders, unique bone status characteristics may result in lower correlations between REMS and DXA, underscoring the need for tailored assessment; (iii) FS, a parameter derived from REMS, shows significant potential in predicting the risk of fragility fractures; (iv) REMS offers good clinical applicability across diverse populations, including those requiring radiation-free monitoring or frequent monitoring; (v) REMS addresses specific limitations of DXA, particularly the overestimation of BMD caused by imaging artifacts; and (vi) the long-term performance of REMS needs to be validated in more extended longitudinal studies involving patients with diverse clinical characteristics.

Notably, REMS has shown comparable diagnostic capabilities to DXA [15], with high sensitivity that does not compromise specificity, as both metrics remained strong across studies. This indicates that REMS is capable of accurately identifying individuals with low bone mineral density while minimizing false positives. However, the exclusion of error-prone REMS reports in some analyses may have influenced specificity, and further studies in diverse clinical settings are needed to confirm these findings in routine practice.

A striking finding is that REMS improves the diagnosis of osteoporosis in cases where DXA’s accuracy is impaired by artifacts such as vertebral fractures or OA [29,37,51]. These conditions, which frequently coexist with osteoporosis [52], alter bone geometry and microarchitecture, potentially complicating fracture prediction [53]. In the spine, OA is characterized by several manifestations including disc space narrowing, facet joint arthropathy, end-plate osteophytosis, and vertebral plate sclerosis [54]. Given that DXA generates a two-dimensional antero-posterior projection of the lumbar spine, these structural abnormalities may superimpose on the measured area, affecting the accuracy of areal BMD evaluations.

While current DXA protocols recommend excluding the vertebrae with significant degenerative changes or T-score differences greater than 1.0, this adjustment has a limited impact on improving fracture prediction [55]. Furthermore, DXA-based lumbar BMD is often artificially increased by aortic calcifications [25], which lateral lumbar DXA imaging can mitigate; however, this approach is rarely used due to technical challenges, patient mobility issues, and the overlapping of anatomical structures. QUS has also been explored as a non-ionizing alternative for bone assessments, but peripheral site measurements and inconsistent results limit its clinical utility [56].

REMS’s accessibility, portability, and non-ionizing nature make it a valuable tool for frail or immobile patients, potentially improving treatment adherence and enabling bedside evaluations. As suggested by the previously mentioned GRADE recommendations deployed by ESCEO, REMS shows good promise in these particular populations, where it can be used as an alternative for osteoporosis diagnosis and post-fracture management [16]. These categories of patients might also include those with CKD, who experience significant bone metabolism abnormalities (e.g., hypocalcemia, vitamin D deficiency, or hyperparathyroidism) that increase fracture risk [25,46]. REMS’s automated software effectively accounts for vertebral shape artifacts, calcifications, and surgical interventions such as kyphoplasty, allowing for more accurate assessments compared to DXA in these cases.

Radiation-sensitive populations, such as pregnant women, children, and patients requiring frequent monitoring, stand to benefit significantly from REMS, which may enable the early detection of conditions such as pregnancy-associated osteoporosis or transient osteoporosis of the hip [57]. Specifically, pregnancy induces substantial changes in maternal calcium metabolism, including heightened osteoblastic and osteoclastic activity, with increased bone demineralization risk [58]. One study observed a 2% decrease in maternal BMD at the FN between the first and the third trimester using REMS [59,60]. Another study by Degennaro et al. reported an 8.1% reduction in mean BMD in pregnant women with respect to non-pregnant controls matched for age, ethnicity, and BMI [61]. Although these changes are typically modest and reversible postpartum, they increase the risk of osteopenia, bone fragility, and in rare cases, osteoporosis. This can be exacerbated in pregnant women already diagnosed with low BMD or with conditions predisposing them to osteoporosis.

Rheumatic diseases warrant consideration in this regard. They encompass a wide range of chronic conditions that primarily affect the musculoskeletal system but also increase the risk of osteoporosis due to chronic inflammation, frequent corticosteroid use, immobilization or reduced physical activity, nutritional deficiencies, and demographics such as age or gender [62]. Comprehensive management of immune-mediated disorders should promote regular osteoporosis screening to address this elevated risk. A cross-sectional study carried out by Bojinca et al. in 2019 [63] evaluated osteoporosis in patients with rheumatoid arthritis (RA) using REMS. The findings indicated that REMS identified lower BMD values, a higher prevalence of osteoporosis, and an increased fracture risk in RA patients compared to controls. However, a comparative assessment between REMS and DXA in these patients was not performed. Strong positive correlations between DXA- and REMS-derived T-scores were revealed in patients with axial spondyloarthritis [64], particularly at TH (r = 0.80, *p* < 0.01) and FN (r = 0.77, *p* < 0.01). However, DXA-measured T-scores at the antero-posterior LS were significantly higher than the results provided by REMS. Enthesitis, spondylitis, bony erosions or formations, ankylosis, syndesmophytes, or discitis are possible factors influencing this particular relationship between DXA and REMS in this condition and need to be considered.

Furthermore, patients with secondary osteoporosis and conditions like type 1 diabetes mellitus, OI, rheumatic diseases, and inflammatory bowel diseases require longitudinal monitoring, often involving repeated imaging [43]. Notably, Caffarelli et al. [19] showed that REMS diagnosed osteoporosis in more cases than DXA, likely due to DXA artifacts such as degenerative changes, vascular calcifications, and vertebral fractures that falsely elevate BMD estimates [65,66]. However, study limitations include a homogeneous sample of elderly women with longstanding T2DM, making it difficult to separate age-related from diabetes-associated bone complications [67], along with the lack of quantification of degenerative lesion severity.

Of particular significance is REMS-derived FS, which characterizes bone microarchitecture independently of BMD and has the potential to address the limitations of existing tools in predicting fracture risk, particularly in patients with normal or only slightly reduced BMD [68]. Emerging evidence supports FS as a predictor of imminent fracture risk, complementing longer-term risk assessments like FRAX. FS also demonstrates low precision errors and is sensitive to subtle skeletal changes [18], suggesting its utility in managing patients with secondary osteoporosis, diabetes, and other conditions where DXA-measured BMD inadequately reflects fracture risk. Further longitudinal studies are needed to validate its reliability and potential for FRAX adjustment [69].

Structured expert feedback has highlighted REMS’ reduced costs as a key factor in its potential for widespread adoption [70]. A cost-minimization analysis conducted within the Italian National Health Service (NHS) revealed lower direct healthcare costs associated with REMS compared to DXA, with savings driven by reduced professional time and fewer additional tests. Also, the cost-effectiveness and economic impact of REMS in osteoporosis diagnosis were evaluated in US women older than 50. This was assessed by estimating the cost per quality-adjusted life year (QALY) for performing REMS followed by treatment vs. delaying the diagnosis and providing late treatment. The results indicated more QALYs, as well as fewer fractures and reduced fracture-related costs, in patients where REMS examination was followed by treatment [71]. This economic advantage further supports REMS as a viable alternative for osteoporosis diagnosis and monitoring [70].

Despite encouraging results, several constraints of this systematic review should be acknowledged. Firstly, a limited number of comparative studies between REMS and DXA exist, reflecting the recent emergence of REMS, and the outcomes may vary across the heterogeneous populations included. Secondly, most available studies are cross-sectional, restricting conclusions about REMS’s long-term reproducibility and utility in diverse clinical backgrounds. Similarly, data on non-Caucasian populations and individuals with a high BMI remain scarce and inconclusive. Furthermore, our search was confined to three databases (Web of Science, Pub Med, and Science Direct) focused solely on English-language literature, and excluded grey literature, which may have impacted the comprehensiveness of the review. Lastly, we opted not to include “DEXA” in our search string, despite its widespread informal use, as the International Society for Clinical Densitometry designates “DXA” as the preferred terminology for the technology [72]. Additionally, “DEXA” is frequently associated with dexamethasone, which could introduce irrelevant search results. While this decision helped maintain search specificity, it may have limited the number of retrieved articles, as some studies may still use “DEXA” in their indexing.

Future research should explore whether REMS can serve as a main diagnostic tool for both primary and secondary osteoporosis and assess its performance across various comorbidities and demographic groups. Additionally, longitudinal studies are required in order to establish the role of fragility score as a reliable fracture risk predictor. Comparative studies with further imaging technologies such as lateral DXA imaging, quantitative computed tomography, or other advanced diagnostic techniques (which are scarcely available at the moment) may strengthen the evidence for REMS.

## 5. Conclusions

This systematic review provides a comprehensive evaluation of REMS as an emerging imaging modality for osteoporosis diagnosis and fracture risk assessment, highlighting its diagnostic performance, clinical utility, and economic implications. The findings indicate that REMS demonstrates diagnostic accuracy comparable to DXA, with high concordance, sensitivity, and specificity. However, in patients with chronic conditions, unique bone status characteristics may influence REMS-DXA correlations, emphasizing the need for tailored assessments.

Furthermore, REMS-derived FS shows strong potential in predicting fragility fractures, offering a valuable tool for improving fracture risk stratification alongside the classical bone parameters.

Beyond its diagnostic capabilities, REMS presents significant clinical advantages, including portability, cost-effectiveness, and the ability to provide radiation-free monitoring. These attributes make it particularly beneficial for radiation-sensitive populations, such as pregnant women, children, and individuals requiring frequent imaging, as well as for frail or mobility-impaired patients. Additionally, REMS addresses specific limitations of DXA, such as the overestimation of BMD due to imaging artifacts from vertebral fractures, osteoarthritis, and vascular calcifications.

Despite these promising attributes, the long-term performance of REMS remains to be fully established. The relatively limited availability of comparative studies with DXA, the predominance of cross-sectional research, and the lack of extensive data on diverse populations, including non-Caucasian individuals and those with high BMI, underscore the need for further investigation. Future studies should explore REMS’ applicability across various comorbidities, validate FS as a reliable fracture risk predictor, and assess its role as a primary diagnostic tool for osteoporosis.

## Abbreviations

The following abbreviations are used in this manuscript:

AN	Anorexia nervosa
AUC	Area under the curve
BMD	Bone mineral density
BMI	Body mass index
CASP	Critical Appraisal Checklists
CKD	Chronic kidney disease
DALYs	Disability-adjusted life-years
DeFra	FRAX- Derived Fracture Risk Assessment
DXA	Dual X-ray absorptiometry
ESCEO	European Society for Clinical and Economic Aspects of Osteoporosis and Musculoskeletal Diseases
FDA	Food and Drug Administration
FEA	Finite Element Analysis
FN	Femoral neck
FRAX	Fracture Risk Assessment
FS	Fragility Score
GRADE	Grading of Recommendations, Assessment, Development, and Evaluations
HR-pQCT	High-resolution peripheral quantitative computed tomography
IOF	International Osteoporosis Foundation
k	Cohen’s kappa coefficient
K-L	Kellgren-Lawrence grading system
LS	Lumbar spine
LSC	Least significant change
MHz	Mega Hertz
MRI	Magnetic Resonance Imaging
OA	Osteoarthritis
OI	Osteogenesis imperfecta
PICO	Population, Intervention, Comparison and Outcome
pQCT	Peripheral quantitative computed tomography
PRISMA	Preferred Reporting Items for Systematic Reviews and Meta-Analysis Checklist
QALY	Quality-adjusted life year
QCT	Quantitative computed tomography
QUS	Quantitative Ultrasonography
REMS	Radiofrequency echographic multispectrometry
RMS-CV	Root-mean-square coefficient of variation
ROIs	Regions of interest
SCI	Science Citation Index
SDs	Standard deviations
T2DM	Type-2 diabetes mellitus
TBS	Trabecular bone score
TH	Total hip
US	Ultrasound
USA	United States of America
WHO	World Health Organization

## Appendix A

**Table A1:** CASP Checklist analysis of the included cross-sectional articles; “+” indicates that the article meets the criteria mentioned above; “-” indicates that the article does not meet the abovementioned criteria; “/” indicates the “can’t tell” option. Abbreviations: BMD—bone mineral density; T2DM—type II diabetes mellitus; PM—post-menopause; LS—lumbar spine; FN—femur neck; DXA—dual-energy X-ray absorptiometry; REMS—radiofrequency echographic multi spectrometry; SG—study group; CG—control group; OA—osteoarthritis; BMI—body mass index; TH—total hip; TBS—trabecular bone score; HR-pQCT—high-resolution peripheral quantitative computed tomography; OP—osteoporosis; p-OP—primary osteoporosis; s-OP—secondary osteoporosis.

First Author, Publication Year	1. Clearly Focused Issue	2. Appropriate Method to Answer the Question	3. Subjects Recruitment	4. Measurements Accurately Performed	5. Appropriate Data Collection	6. Sample Size	7. Results Presentation	8. Data Analysis	9. Statement of Findings	10. Applica-bility of the Results to Local Population	11. Value of Research	**Positive Appraisal**	**Negative Appraisal**	**Unknowns**
Adami et al., 2024 [27]	+	+	+	+	+	+	Sensitivity and specificity of REMS in diagnosis of osteoporosis are above 90%.	+	+	+	+	Multi-center design, rigorous adherence to guidelines.	Only Caucasian males, no patients with secondary osteoporosis.	DXA was performed at only one site in each patient.
Amorim et al., 2021 [23]	+	+	+	+	+	/	The REMS approach had high accuracy for the diagnosis of osteoporosis in comparison with DXA in adult women.	+	+	+	+	First study on REMS in a Latin American population.	High number of REMS scans excluded;	Real-life experience (multiracial, broad age range, osteo-degenerative processes were maintained in DXA)
Caffarelli et al., 2022 [19]	+	+	-	+	+	+	REMS-BMD values were lower in women with T2DM than in controls.	+	+	+	+	Clear research focus; well-defined inclusion/exclusion criteria.	No power calculation for sample size; cross-sectional; findings limited to elderly PM women with longstanding T2DM.	Limited discussion on potential confounding factors.
Caffarelli et al., 2022 [37]	+	+	+	+	+	+	In OA subjects, more women were classified as “osteoporotic” on the basis of BMD by REMS with respect to those classified by DXA.	+	+	+	+	Well-defined cohort, strict inclusion/exclusion criteria, subgroup analysis	Relatively small sample size, lack of third reference technique.	Grade of OA in the hip, exclusion of severely obese patients.
Caffarelli et al., 2024 [29]	+	+	+	+	+	+	REMS proved a higher capability to diagnose OP compared to DXA in a population with varying severity levels of OA.	+	+	+	+	Large sample, 2 radiologists evaluating the OA grade, DXA and REMS performed by 2 experienced operators.	Exclusively Caucasian patients with a BMI 18.5–39.9 kg/m^2^.	Joint space narrowing and osteophytes not separately assessed.
Caffarelli et al., 2022 [41]	+	+	+	+	+	-	A good correlation was detected between BMD obtained by DXA and REMS at LS, FN, TH.	+	+	+	+	Control group; all instrumental investigations performed by 2 operators.	Small sample, cross-sectional.	Comorbidities; therapies
Caffarelli et al., 2023 [24]	+	+	+	+	+	-	BMD-DXA and REMS had good correlation at all sites; significant association between REMS and TBS.	+	+	+	+	Control group; all DXA and REMS scans carried out by a single operator.	Small sample size.	Lack of evaluation using HR-pQCT.
Cortet et al., 2021 [21]	+	+	+	+	+	+	The diagnostic effectiveness of REMS was confirmed in a large series of female patients of all ages.	+	+	+	+	Multicentric, broad age range for subgroup analysis, detailed statistics.	Female patients only.	Lack of a follow-up and analysis of the occurrence of incident fragility fractures.
Di Paola et al., 2019 [15]	+	+	+	+	+	+	REMS- BMD showed a good agreement with DXA-BMD in both LS and hip.	+	+	+	+	Large sample size; first study evaluating the performance of REMS compared to DXA.	The number of REMS reports excluded; population entirely of Caucasian women.	BMD by the Lunar Prodigy densitometers were converted in Hologic equivalent values.
Fassio et al., 2024 [46]	+	+	+	+	+	-	DXA provided higher BMD values at the lumbar spine than REMS.	+	+	+	+	Addressed the gap represented by CKD patients.	Limited sample, absence of a control group.	Lack of evaluation using HR-pQCT.
Fassio et al., 2022 [25]	+	+	+	+	-	-	Diagnostic agreement between DXA and REMS was low for LS, moderate for FN and significant for TH.	+	+	+	+	Real-life explorative study; Aortic calcifications studied on DXA images.	Limited sample, absence of a control group.	Only a minority of the patients showed increased serum levels of PTH.
Ishizu et al., 2024 [35]	+	+	+	+	+	-	REMS may overcome the overestimation of BMD by DXA due to vertebral deformities, abdominal aortic calcification, and DM	+	+	+	+	Homogeneous cohort;	Single-center study, no separation of p-OP/s-OP, only unilateral femoral BMD scans; pattern for prescription of medications	Ossification of the spinal ligaments and presence of osteophytes were not assessed.
Lalli et al., 2022 [26]	+	+	+	+	+	-	Statistically significantdifferences in the fragility score were obtained between the fractured and non-fractured patients for both populations.	+	-	+	+	Presence of a control group with p-OP, extensive statistical analysis.	Cross-sectional, number of patients with disuse-related OP relatively small.	BMD by the Lunar Prodigy densitometers were converted in Hologic equivalent values.
Nowakowska-Plaza et al., 2021 [7]	+	+	+	+	+	/	Strong correlations between REMS and DXA results were found in both groups, regardless of the sex, age, and BMI.	+	+	+	+	Rigorous statistical analysis; Sex, age, BMI- subgroup analysis	Relatively small sample size;	Limited discussion on potential confounding factors, such as other comorbidities or lifestyle factors.
Rolla et al., 2020 [36]	+	+	+	+	+	-	Weak positive correlations between LS, FN DXA and REMS T-scores were observed in the SG.	-	-	+	+	Control group; Subgroup analysis for SG.	Small sample size; lack of patients with active disease in SG; no DXA scans in the CG; no objective assessment of fractures.	Various durations of the disease; time span of therapy; Calcium and vitamin D supplementation not recorded.

**Table A2:** CASP Checklist analysis of the included cohort studies; “+” indicates that the article meets the criteria mentioned above; “-” indicates that the article does not meet the abovementioned criteria; Abbreviations: BMD—bone mineral density; FS—fragility score; REMS—radiofrequency echographic multi spectrometry.

First Author, Publication Year	1. Focused Issue	2. Subjects Recruitment	3. Measurement of Exposure	4. Measurement of Outcome	5a. Identification of All Important Confounding Factors	5b. Assessment of All Confounding Factors Specified	6a. Complete Follow-Up	6b. Length of Follow-Up	7. Results	8. Precision of Results	9. Credibility of the Results	**10. Application of Results to Local Population**	**11. Compatibility with Other Studies**	**12. Implication for Study Practice**	**Positive Appraisal**	**Negative Appraisal**
Adami et al., 2020 [22]	+	+	+	+	+	-	+	+	REMS T-score resulted an effective predictor for the risk of incident fragility fractures in a female cohort.	+	+	+	+	+	Age-matched groups; prospective cohort.	Non-specific enrolment criteria, the main risk factors for fragile fractures not considered, other parameters of bone strength except for BMD not considered.
Pisani et al., 2023 [68]	+	+	+	+	+	-	+	+	FS showed the highest prediction ability for any fracture type in both genders.	+	+	+	+	+	Prospective study, large sample.	Clinical factors predicting risk of fractures not considered.

## Appendix B

**Table A3:** Summary of key features from reviewed articles. Abbreviations: SG—study group; CG—control group; F—female; M—male; LS—lumbar spine; FN—femur neck; TH—total hip; NR—not reported; DXA—dual-energy X-ray absorptiometry; dg—diagnosed; OP—osteoporosis; T2DM—type II diabetes mellitus; BMI—body mass index; dep.—depending; OA—osteoarthritis; VF—vertebral fractures; FS—fragility score; OI—osteogenesis imperfecta; K-L score—Kellgren–Lawrence score; p-OP—primary osteoporosis; d-r OP—disuse-related osteoporosis.

First author, Publication Year, Country	Study Design	Sample Size	Participant Description	Mean Age	Gender	Race/Ethnicity	DXA Machine Type; Evaluated Site	REMS/DXA Diagnosis Accuracy LS; FN		***p*-Value**	**Confounding Factors**
Adami et al., 2020, Italy [22]	Prospective cohort	1370 (192 SG; 1178 CG)	Women between 30–90 years	Subgroup analysis	F	Caucasians	Hologic;LS; FN	Sensitivity LS; FN	65.1%; 40.2% for fragility fractures	<0.001	Vertebral fractures
								Specificity LS; FN	57.1%;79.9% for fragility fractures	<0.001	
								Diagnostic concordance (k=) LS; FN	0.8; NR (OP dg agreement 84.8%, 84.2%)	<0.001	
								Correlation (r=) LS; FN	0.92	<0.001	
Adami et al., 2024, Italy [27]	Cross-sectional	508 LS; 512 FN	Men with a medical prescription for DXA	58.3 ± 14.5 LS; 58.9 ± 14.4 FN	M	Caucasians	NR; LS, FN	Sensitivity LS; FN	90.1%; 90.9%	<0.05	Age, BMI
								Specificity LS; FN	93.6%; 94.6%	<0.05	
								Diagnostic concordance (k=) LS; FN	0.71; 0.71	<0.001	
								Correlation (r=) LS; FN	0.91; 0.90	<0.0001	
Amorim et al., 2021, Brazil [23]	Cross-sectional	343	Women 30–80 years old	59.9 ± 10.2	F	Asians 8.4%, Caucasians 69.6%, Miscigenated 7.1%	Hologic; LS; FN	Sensitivity LS; FN	80%; 85%	NR	Age, ethnicity. BMI, comorbidities
								Specificity LS; FN	94%; 93%	NR	
								Diagnostic concordance (k=) LS; FN	0.47; 0.53	<0.001	
								Correlation (r=) LS; FN	0.75; 0.78	<0.001	
Caffarelli et al., 2022, Italy [37]	Cross-sectional	159 (113 OA; 46 VF)	Postmenopausal women with vertebral OA/fractures.	63.2 ± 11.3 OA; 73.6 ± 18.5 VF	F	Caucasians	Hologic; LS, FN, TH	Sensitivity LS; FN	NR	NR	Age, BMI, menopause
								Specificity LS; FN	NR	NR	
								Diagnostic concordance (k=) LS; FN	NR (total REMS/DXA OP dg: 35.1%, 9.3%)	<0.05	
								Correlation (r=) LS; FN	NR	NR	
Caffarelli et al., 2022, Italy [41]	Cross-sectional	77 (47 SG; 30 CG)	Adolescent and young women, BMI < 18 kg/m^2^	31.7 ± 10.3 (SG); 32.9 ± 9.5 (CG)	F	Caucasians	Hologic; LS, FN, TH	Sensitivity LS; FN	NR	NR	Disease duration
								Specificity LS; FN	NR	NR	
								Diagnostic concordance (k=) LS; FN	NR	NR	
								Correlation (r=) LS; FN	0.64; 0.86	<0.01	
Caffarelli et al., 2024, Italy [29]	Cross-sectional	431	Patients with OA at the LS.	63.9 ± 11.2	F, M	Caucasians	Hologic; LS; FN	Sensitivity LS; FN	NR	NR	Osteophytes, space narrowing
								Specificity LS; FN	NR	NR	
								Diagnostic concordance (k=) LS; FN	NR; (REMS/DXA dg OP: 52%, 34.5%)	*p* < 0.001	
								Correlation (r=) LS; FN	0.98, 0.85, 0.45 for LS with K-L score of 0/1, 2, 3	<0.0001	
Caffarelli et al., 2022, Italy [19]	Cross-sectional	175 (88 SG; 87 CG)	Postmeno-pausal	70.5 ± 7.6 (SG); 69.2 ± 7.5 (CG)	F	Caucasians	Hologic; LS, FN, TH	Sensitivity LS; FN	NR	NR	T2DM, age, BMI, smoking, postmenopausal status
								Specificity LS; FN	NR	NR	
								Diagnostic concordance (k=) LS; FN	NR; (total REMS/DXA dg OP: 47%, 28%)	<0.05	
								Correlation (r=) LS; FN	NR	NR	
Caffarelli et al., 2023, Italy [24]	Cross-sectional	77 (41 SG; 36 CG)	Subjects with clinical or genetic diagnosis of OI type I, III or IV.	40.5 ± 18.7 SG, 41.7 ± 16.3 CG	F, M	Caucasians	Hologic; LS; FN	Sensitivity LS; FN	NR	NR	Calcium, vitamin D supplementation
								Specificity LS; FN	NR	NR	
								Diagnostic concordance (k=) LS; FN	NR	NR	
								Correlation (r=) LS; FN	0.35; 0.54	<0.01	
Cortet et al., 2021, Spain, Belgium, UK, Italy [21]	Cross-sectional	4307	Female patients between 30–90 years	Subgroup analysis	F	Caucasians	Hologic; LS; FN	Sensitivity LS; FN	90.4% overall	<0.05	Menopause, age
								Specificity LS; FN	95.5% overall	<0.05	
								Diagnostic concordance (k=) LS; FN	0.83 overall	<0.05	
								Correlation (r=) LS; FN	0.95; 0.93	<0.05	
Di Paola et al., 2019 [15]	Cross-sectional	1914 (1553 LS; 1637 FN)	Postmenopausal women	60.7 ± 5.4 LS; 60.9 ± 5.5 FN	F	Caucasians	Hologic or Lunar GE; LS; FN	Sensitivity LS; FN	91.7%; 91.5%	<0.05	Menopause, age
								Specificity LS; FN	92%; 91.8%	<0.05	
								Diagnostic concordance (k=) LS; FN	0.824; 0.794	<0.001	
								Correlation (r=) LS; FN	0.94; 0.93	<0.001	
Fassio et al., 2024 [46]	Cross-sectional	40	Subjects underwent kidney transplantation	60.43 ± 9.8	F, M	Caucasians	GEN Lunar; LS; FN	Sensitivity LS; FN	NR	NR	Fractures were not objectively assessed
								Specificity LS; FN	NR	NR	
								Diagnostic concordance (k=) LS; FN	NR	NR	
								Correlation (r=) LS; FN	0.4; 0.7	<0.01; <0.0001	
Fassio et al., 2022, Italy [25]	Cross-sectional	41	Patients undergoing peritoneal dialysis	61.1 ± 13.7	F, M	Caucasians	GE Lunar; LS; FN	Sensitivity LS; FN	NR	NR	Aortic calcifications
								Specificity LS; FN	NR	NR	
								Diagnostic concordance (k=) LS; FN	0.321; 0.445	0.026; <0.01	
								Correlation (r=) LS; FN	NR	NR	
Ishizu et al., 2023, Japan [35]	Cross-sectional	70	Patients whoreceived osteoporosis treatment and BMD assessment byDXA.	78.39 ± 9.50	F, M	Asians	GE Lunar; LS, FN	Sensitivity LS; FN	NR	NR	Age, comorbidities vertebral fractures, hip OA, aortic calcifications
								Specificity LS; FN	NR	NR	
								Diagnostic concordance (k=) LS; FN	NR; NR (total dg OP REMS/DXA: 90%; 72.9%)	NR	
								Correlation (r=) LS; FN	0.471; 0.361	<0.001	
Lalli et al., 2022, Italy [26]	Cross-sectional	175 (140 p-OP; 35 d-r OP)	Patients recruited from the rehabilitation units of 5 hospitals.	74 p-OP; 57 d-r OP	F, M	Caucasians	Hologic or GE Lunar; FN, TH	Sensitivity LS; FN	NR	NR	Age, sex, BMI, major osteoporotic fractures
								Specificity LS; FN	NR	NR	
								Diagnostic concordance (k=) LS; FN	NR; 0.31 for p-OP; −0.04 for d-r OP	0.0001	
								Correlation (r=) LS; FN	NR; NR	NR	
Nowakowska-Plaza et al., 2021, Poland [7]	Cross-sectional	116	Men and women with medical indication for DXA.	Range 40–87 years	F, M	Caucasians	Hologic; LS; FN	Sensitivity LS; FN	NR	NR	Age, Menopause
								Specificity LS; FN	NR	NR	
								Diagnostic concordance (k=) LS; FN	0.611; 0.667	<0.05	
								Correlation (r=) LS; FN	0.867; 0.871	<0.001	
Pisani et al., 2023, Italy [68]	Prospective cohort	1804 LS; 1679 FN	Patients had no significant walking impairments.	Subgroup analysis	F, M	Caucasians	NR; LS, FN	Sensitivity LS; FN	72.4%; 70% for FS in F; 71.6%, 72.2% for FS in M	<0.001	Age, BMI, Menopause, comorbidities
								Specificity LS; FN	77.9%; 73.2% for FS in F; 79%; 76.1% for FS in M	<0.001	
								Diagnostic concordance (k=) LS; FN	NR	NR	
								Correlation (r=) LS; FN	NR	NR	
Rolla et al., 2020, Poland [36]	Cross-sectional	57 (33 SG; 24 CG)	Well-controlled and surgery-cured acromegaly	59.1 ± 9.8 (SG); 55.5 (CG)	F, M	Caucasians	Hologic; LS, FN	Sensitivity LS; FN	NR	NR	Acromegaly, DM, hypogonadism, age, menopause
								Specificity LS; FN	NR	NR	
								Diagnostic concordance (k=) LS; FN	NR	NR	
								Correlation (r=) LS; FN	0.482; 0.431	0.011; 0.018	
